# Relationship Between Media Coverage and Measles-Mumps-Rubella (MMR) Vaccination Uptake in Denmark: Retrospective Study

**DOI:** 10.2196/publichealth.9544

**Published:** 2019-01-23

**Authors:** Niels Dalum Hansen, Kåre Mølbak, Ingemar Johansson Cox, Christina Lioma

**Affiliations:** 1 Statens Serum Institut Copenhagen Denmark; 2 Department of Computer Science University of Copenhagen Copenhagen Denmark; 3 Department of Computer Science University College London London United Kingdom

**Keywords:** online news media, vaccination uptake, media influence on vaccination uptake, MMR, autism

## Abstract

**Background:**

Understanding the influence of media coverage upon vaccination activity is valuable when designing outreach campaigns to increase vaccination uptake.

**Objective:**

To study the relationship between media coverage and vaccination activity of the measles-mumps-rubella (MMR) vaccine in Denmark.

**Methods:**

We retrieved data on media coverage (1622 articles), vaccination activity (2 million individual registrations), and incidence of measles for the period 1997-2014. All 1622 news media articles were annotated as being provaccination, antivaccination, or neutral. Seasonal and serial dependencies were removed from the data, after which cross-correlations were analyzed to determine the relationship between the different signals.

**Results:**

Most (65%) of the anti-vaccination media coverage was observed in the period 1997-2004, immediately before and following the 1998 publication of the falsely claimed link between autism and the MMR vaccine. There was a statistically significant positive correlation between the first MMR vaccine (targeting children aged 15 months) and provaccination media coverage (*r*=.49, *P*=.004) in the period 1998-2004. In this period the first MMR vaccine and neutral media coverage also correlated (*r*=.45, *P*=.003). However, looking at the whole period, 1997-2014, we found no significant correlations between vaccination activity and media coverage.

**Conclusions:**

Following the falsely claimed link between autism and the MMR vaccine, provaccination and neutral media coverage correlated with vaccination activity. This correlation was only observed during a period of controversy which indicates that the population is more susceptible to media influence when presented with diverging opinions. Additionally, our findings suggest that the influence of media is stronger on parents when they are deciding on the first vaccine of their children, than on the subsequent vaccine because correlations were only found for the first MMR vaccine.

## Introduction

Reaching all children with two doses of a measles vaccine is an important aim of all national immunization programs. However, many countries have difficulties achieving the declared aim of measles elimination [1]. Achieving and maintaining measles elimination is possible through a two-dose vaccination program with vaccination coverage of at least 95% for both doses [2]. According to World Health Organization statistics for 2016, only 41/160 (25.6%) countries have a coverage of 95% for the second measles-mumps-rubella (MMR) vaccine [3]. A 2009 assessment of measles elimination in Europe attributed differences in measles incidence in European countries to the varying degrees of success of the national immunization programs [1] (ie, lower coverage equals higher incidence). Several factors influence the success of an immunization program, including accessibility and availability of vaccination clinics, knowledge about vaccination-preventable diseases, and vaccination cost [4]. In this study, we aim to investigate the relationship between media coverage, the incidence of measles, and the vaccination uptake for the MMR vaccine in Denmark. For our analysis, we use historical data on vaccination and media activity over an 18-year period (January 1, 1997, to December 31, 2014).

The safety of the MMR vaccine became an important topic after 1998 when Wakefield [5] falsely claimed a link between the MMR vaccine and autism. This reduced the public confidence in the vaccine and resulted in a drop in vaccination uptake from above 90% to 79% in England [6,7]. The uptake of the MMR vaccine has also in Denmark been vulnerable to negative media attention. In 1993, the safety of the vaccine was questioned in a nationwide TV program, resulting in record low vaccination coverage [8]. This vulnerability of a vaccination program to public distrust is not limited to the MMR vaccine. Recently the fear of adverse reactions to the human papillomavirus vaccine caused a significant decline in vaccination uptake in Denmark [9]. Hypothesizing that there is a link between media coverage and changes in vaccination coverage is not new [10-13]. However, no study has examined the relationship over an extended period. Understanding the relationship between media coverage and vaccination uptake may underpin the design of public health communication strategies and the development of new surveillance strategies.

In this paper, we take advantage of an 18-year long time series to analyze the correlation between MMR vaccinations, the incidence of measles and media coverage in Denmark. Additionally, we look at the effect of provaccination versus antivaccination media coverage.

## Methods

### Register Data: Vaccination and Measles Incidence

The MMR vaccination program was introduced in Denmark on January 1, 1987 [14]. The vaccination program consists of 2 vaccinations: 1 targeted at 15-month-old children (MMR-1), and another targeted at 12-year-old children (MMR-2). Since April 1, 2008, the MMR-2 vaccination schedule has changed to target 4-year-old children [15]. Every time a general practitioner vaccinates a child, the date and civil registry number (CRN) of the child are recorded in order for the doctor to receive a reimbursement [16]. These reports are saved in the childhood vaccination database, an immunization information system containing reports from 1997 onwards [16]. Using the CRN, we looked up the birthday of the vaccinated person and calculated the child’s age when receiving the vaccine. We separated the registered MMR vaccines into groups based on the recommended vaccination schedule of 15 months, 4 years or 12 years. Each registered vaccine was assigned to the group where the age of the child at vaccination was closest to the target age of the group. We excluded data on the 4-year-old children because they were not represented in the full study period (this corresponds to 374,867 vaccinations). Table 1 shows a summary of the number of registered vaccinations.

We defined vaccination activity as 100 times the number of children vaccinated in a given month divided by the number of eligible children (ie, for MMR-1 the number of children turning 15 months that month). We controlled for yearly and seasonal variations in vaccination activity by dividing by the birth cohort size.

**Figure 1:**
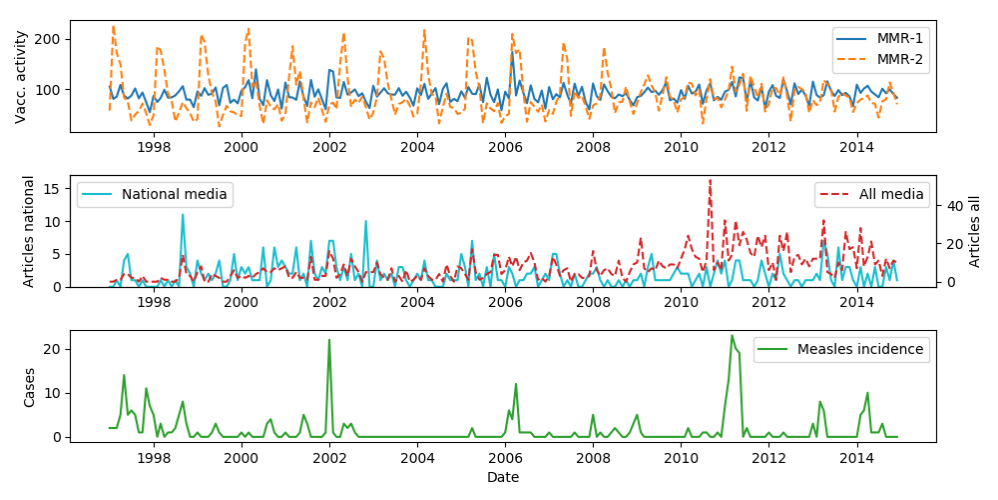
A plot of monthly vaccination activity, media coverage, and measles incidence.

The reported dates of vaccination contain some errors, mainly when doctors report the date of the reimbursement claim instead of the vaccination date. We, therefore, aggregated data on a monthly basis. The top plot in Figure 1 shows the monthly vaccination activity for the 2 vaccines.

To evaluate to what extent MMR vaccination numbers and media coverage about MMR were correlated with the number of measles cases, we also retrieved information about the number of reported measles cases during the study period. Measles is a notifiable disease, and each case is reported to Statens Serum Institut. We aggregated data on a monthly basis, which is shown in Figure 1 (bottom plot). Table 1 shows the total number of reported measles cases in the study period.

**Table 1:** Summary of the study data. Measles-mumps-rubella (MMR) vaccinations were grouped into MMR-1 (15-month-old children) and MMR-2 (12-year-old children).

Variable		Value
**MMR-1**		
	Number of vaccinations	1,098,389
	Age (years) at vaccination, mean (SD)^a^	1.7 (1.4)
**MMR-2**		
	Number of vaccinations	1,108,205
	Age (years) at vaccination, mean (SD)^a^	12.3 (1.8)
**Reported measles cases**		
	Number of cases	334
**Media coverage^b^**		
	All media, N	1622
	National media, n (%)	390 (24.0)
**Analysis of all media content**		
	Relevant to MMR^c^, N	681
	Provaccination, n (%)	430 (63.1)
	Neutral, n (%)	500 (73.4)
	Antivaccination, n (%)	72 (10.6)

^a^The dataset for MMR-1 (targeted 15-month-old children) and MMR-2 (targeted 12-year-old children) only contains age at vaccination in years. This should be considered when interpreting the mean age (SD).

^b^Media coverage is quantified as the number of news items containing MMR related keywords over the 18-year study period.

^c^This row denotes the number of news items that have been labeled as either provaccination, neutral or antivaccination. A news item can get more than one label; hence, the numbers do not sum to 681.

### Web-Mined Data: Media Coverage of Measles-Mumps-Rubella

To determine media coverage of MMR, we used the Infomedia archive [17], an online Danish news archive. The archive covers 9 major Danish newspapers, as well as a variety of other news sources. The number of sources indexed is continuously expanding as local newspapers, magazines, news agencies, web media, radio news, and TV news are added to the archive [17]. Radio news and TV news are included in the archive as written summaries.

To measure media coverage related to the MMR vaccine, we constructed a query to retrieve relevant news items from the Infomedia archive (this is standard practice when mining health information from the web [18,19]). The query was designed to have high sensitivity, in other words, most relevant news items should be retrieved. The high sensitivity came with a loss of specificity, since all articles that merely mentioned the MMR vaccine would be retrieved. The query, which we will refer to as the MMR-query, was:

((“mæslinger” OR “mæslinge” OR “fåresyge” OR “røde hunde” OR “mfr”) AND “vaccine”) OR “mæslingevaccine” OR “fåresygevaccine” OR “røde hunde-vaccine OR mfr-vaccine

where “mæslinger” is the Danish word for measles, “fåresyge” means mumps, “røde hunde” means rubella, and “mfr” is the Danish abbreviation for MMR. This query retrieved all news items mentioning “mæslinger” or “mæslinge” (plural or singular) or “fåresyge” or “røde hunde” together with “vaccine,” or news items where either one of the compound phrases (as shown in the second line of the MMR-query) was present. We did not add search terms regarding vaccination, on the assumption that relevant news items will also mention the vaccine. After retrieval, we counted the number of news items returned for this query, for each month of our study period. This type of analysis, which is based on frequency counts, is inspired by computational epidemiology approaches that use web search frequencies to predict health events (eg, influenza-like illness [20], vaccination coverage [21], or antimicrobial drug consumption [22]).

The Infomedia archive has expanded throughout the 18-year study period. In 1997 the archive indexed news items from 20 sources, while in 2014 this number was 1389. As the number of news sources increased, the number of news items added to the archive each month also increased. To accommodate for this change in archive size, we applied 2 sets of frequency counts: (1) 8 major nationwide newspapers that were present in the full duration of the study and (2) all news sources in the archive. We refer to approach (1) as national media and (2) as all media. The middle plot in Figure 1 shows the monthly number of news items retrieved using the MMR-query for each approach, and Table 1 shows the total number of retrieved news items for the 18-year period.

#### Annotation of News Items

The MMR-query was designed with high sensitivity and low specificity. All retrieved news items were subsequently annotated as being relevant to MMR vaccination or irrelevant to improve the specificity. In addition, relevant news items were labeled as having either provaccination, antivaccination, and neutral stance towards the vaccine. The 3 labels are defined in Textbox 1.

Relevant news items were categorized into 1 or more of the three categories. For example, an article with an interview of an antivaccination group accompanied by comments from a doctor explaining the medical reasons and benefits of getting vaccinated would be categorized as both pro and antivaccination. News items whose main focus was not the MMR vaccine (eg, vaccines for pets, annual accounts of vaccination producers, charities for developing countries) would be viewed as irrelevant and would not be categorized.

### Data Analysis

The data described above is a time-series (ie, it consists of MMR/media signals that have timestamps). We analyzed this data as follows. First, we removed any seasonality to avoid general seasonal trends biasing the results. Second, we quantified the relationship between the MMR and media (or measles) signals.

#### Adjusting for Seasonal Correlations

**Figure 2:**
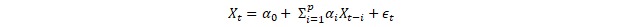
Autoregressive model for x at time t with p autoregressive terms.

In the analysis, we are not interested in effects due to seasonality. For example, reduced vaccination activity during Christmas. Any seasonality or serial dependencies in the signals were therefore removed by fitting an autoregressive model to the signal and subsequently using the residual of the fitted model. An autoregressive model is defined in Figure 2 where where *x* is a time series, *t* is a time point, *p* is the number of autoregressive terms, the *α* is the model coefficient, and *ε*_*t*_ is the residual at time *t*.

**Figure 3:**
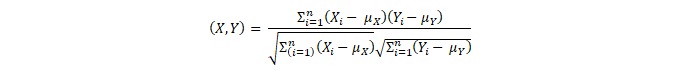
The Pearson r for time series x and y, with mean µ and length n.

To quantify seasonality and serial dependencies we calculated the autocorrelation and partial autocorrelation [23] for all signals. Autocorrelation refers to calculating the Pearson correlation (Pearson r) between the signal and a lagged version of itself. The Pearson correlation for 2 time series, *x* and *y*, with mean *μ* and length *n* is defined in Figure 3.

**Annotation criteria for the news items.**

Provaccination

- News items expressing positive views about the vaccine
- Encouraging people to get vaccinated

Antivaccination

- News items expressing negative views about the vaccine
- Discouraging people to get vaccinated

Neutral

- Neutral information about the vaccine (eg, reports on the number of people vaccinated per year or diseases covered by the vaccine)

The partial autocorrelation can be used to determine the value of *p* in Figure 2 because as the partial autocorrelation approaches zero, the value of additional autoregressive terms is reduced. The partial autocorrelation consists of calculating the correlation between the signal *x*, and a version of itself with a lag of *k* (ie, *x*_*k*_), while at the same time controlling for the autocorrelation of the *k–1* previous lags [23]. The partial autocorrelation at lag *k* can be calculated by fitting an autoregressive model, as defined in Figure 1, with *k* autoregressive terms. The value of the *k*^th^ coefficient (ie, *α*_*k*_, corresponds to the partial autocorrelation at lag *k*).

#### Quantifying the Relationship Between Signals

To quantify the relationship between 2 signals we estimated the cross-correlation. The cross-correlation consists of calculating the Pearson correlation (Figure 3) between 2 signals using different lags. We applied lags between –12 and +12 (ie, up to one year before and after). A cross-correlation of 1 means perfect positive correlation, while a correlation of –1 corresponds to perfect negative correlation. To measure the significance of the correlations we treated a series of *n* cross-correlations as random variables from a student *t* distribution with degrees of freedom *n–1.* We only reported results for the lags where the correlation was significant (ie, *P*<.01).

#### Quantifying the Quality of the News Item Annotation

The first author of this paper (NDH) annotated all news items. A random subset of 200 news items was annotated by a second annotator (the second annotator has no medical or computer science background and works as a legal advisor) to assess the reliability of the annotation. The interannotator agreement is quantified by calculating the Cohen kappa coefficient (κ) [24], which measures interannotator reliability while taking into account chance agreement. The coefficient ranges from –1 to 1, with a common interpretation for κ being that <0 is poor agreement, 0 to .20 is slight, .21 to .40 is fair, .41 to .60 is moderate, .61 to .80 is substantial and .81 to 1.00 almost perfect [25]. Because the 3 categories are not mutually exclusive (ie, a news item can be categorized as both neutral and antivaccination) a kappa coefficient is calculated for each label.

#### Software

The Python packages StatsModels (version 0.8.0) and SciPy (version 0.19.0) were used for calculating autocorrelation, partial autocorrelation, and cross-correlation.

## Results

### Modeling Expected Variations

**Figure 4:**
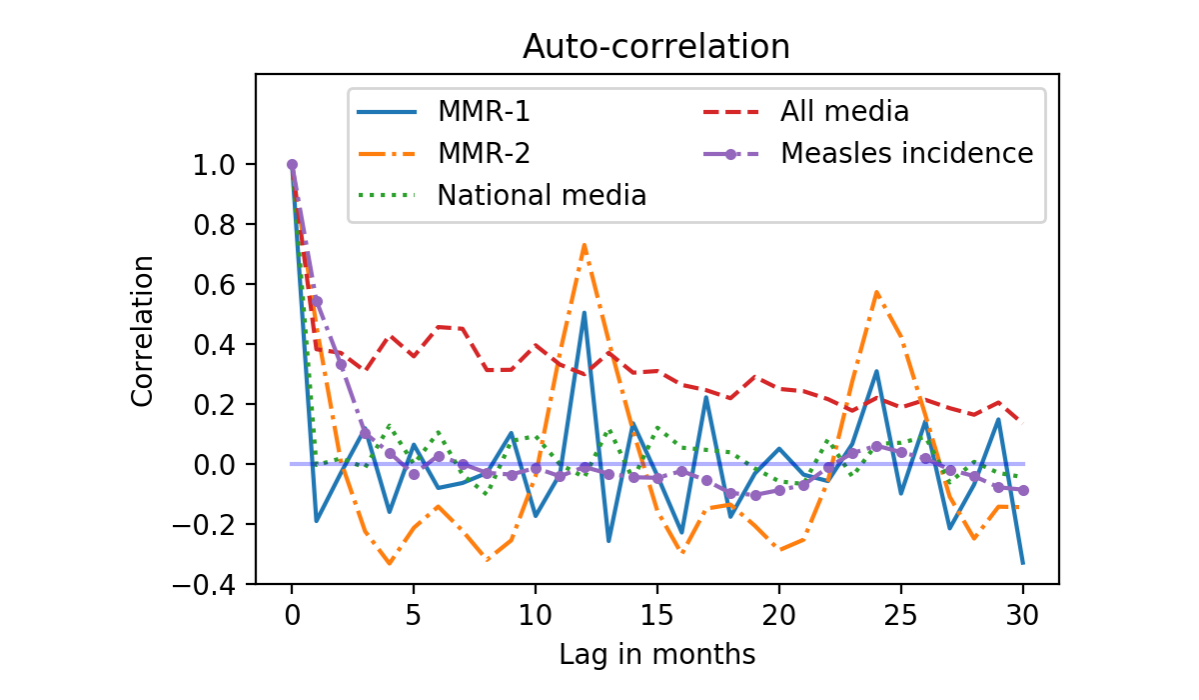
Autocorrelation for MMR-1, MMR-2, national media coverage, all media coverage, and measles incidence. (MMR: measles-mumps-rubella).

Figure 1 (top plot) shows the monthly vaccination activity for MMR-1 and MMR-2. There was a marked periodicity of the number of MMR-2 vaccinations and a visible change in vaccination pattern around 2009. From 1997-2008, inclusive, a reminder letter was sent at the beginning of the year to all children turning 12 that year. The letter was sent at the beginning of each year, and we assume that this was responsible for the annual peak around March. Since May 2014 a reminder letter was sent at age 2, 6.5, and 14 if a child was missing a vaccination [26]. The letters were sent every month, and the effect would, therefore, be spread evenly throughout the year. Figure 4 shows the autocorrelation of vaccination activity, national media (390 articles), all media (1622 articles), and measles incidence. Based on this plot we see that the vaccination activity has a peak at 0, 12, and 24 months. This shows that vaccination activity repeats an annual pattern. For MMR-2 this annual autocorrelation was more pronounced than for MMR-1, likely caused by the pronounced yearly peaks from 1997-2008. For all media, we observed consistent high autocorrelation due to a steady increase in media coverage throughout the study period in the number of retrieved news items. Since this increase was not observed for the national media, the increase is likely explained by the increasing number of media sources in the Infomedia archive, as opposed to generally increased media attention towards the MMR vaccine.

**Figure 5:**
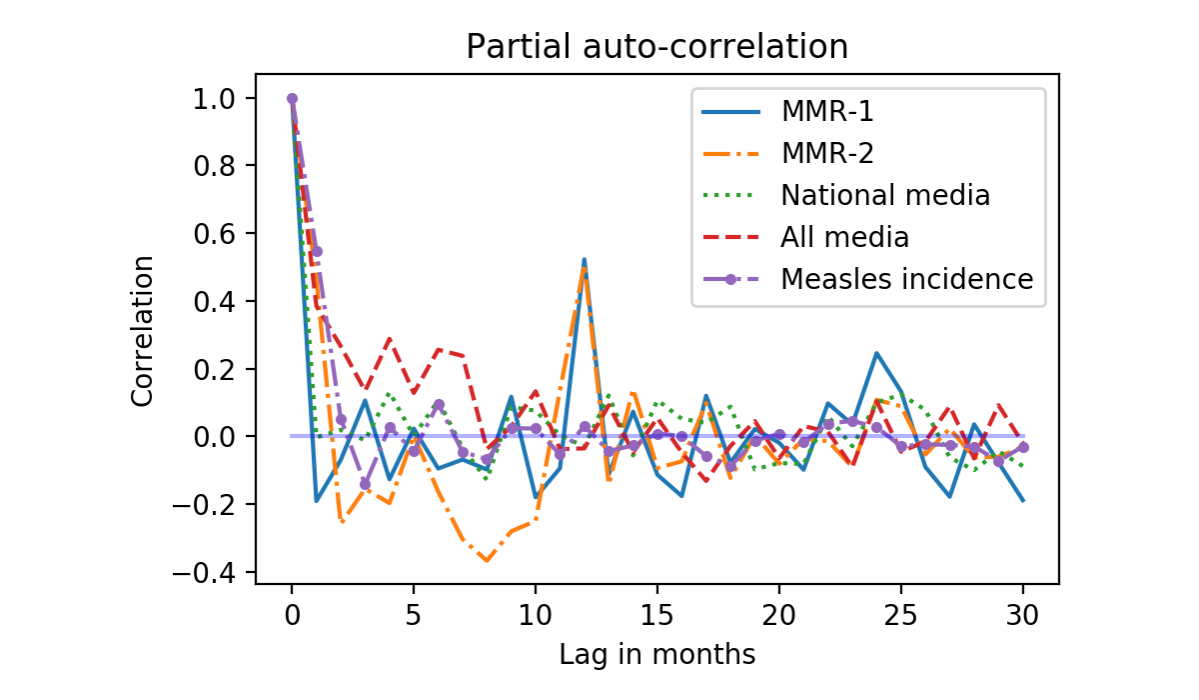
Partial autocorrelation for MMR-1, MMR-2, national media coverage, all media coverage, and measles incidence. (MMR: measles-mumps-rubella).

Figure 5 shows the partial autocorrelation for the vaccination activity, media coverage, and measles incidence. The partial autocorrelation reflects the number of autoregressive terms (ie, *p* in Figure 2) in the autoregressive models used to control for seasonality and serial dependencies. The partial autocorrelation for MMR-1 and MMR-2 quickly drops after the first lag and subsequently peaks again at a 12 months lag. For all media*,* partial autocorrelation remains close to .2 until a 7-month lag after which it fluctuates around zero.

Based on the observation above we applied an autoregressive model with 12 terms, corresponding to the peak in partial autocorrelation for the vaccination activity. We fit the model both to the vaccination activity and to the media coverage time series. The residual (ie, *ε*_*t*_ from Figure 2) will be used in the remaining analysis since this part of the signal is not accounted for by seasonality or serial dependencies.

Figure 5 shows that when an autoregressive model with 12 terms was fitted to the all media signal, then seasonal dependencies are removed. To assess to what extent this was also the case for a general upwards or downwards trend, we fitted a linear model with only a trend term and intercept to the all media signal and the residual of the autoregressive model. For the original signal, the trend was 0.0672 with *P*<.001, while for the residual the trend was 0.0072 with *P*=.32. In other words, with a 0.0672 monthly increase in the number of news items over 18 years, we would expect to see 14.5 additional news items in the last month of the period compared to the first. While for the residual this increase is only 1.6 news items over an 18-year period. Because controlling for seasonal dependencies also removed the bias from a general upwards trend in the media coverage, we will, for brevity, in the remainder of the article focus on the results from all media, and disregard the results using national media.

### Annotation of News Items

**Figure 6:**
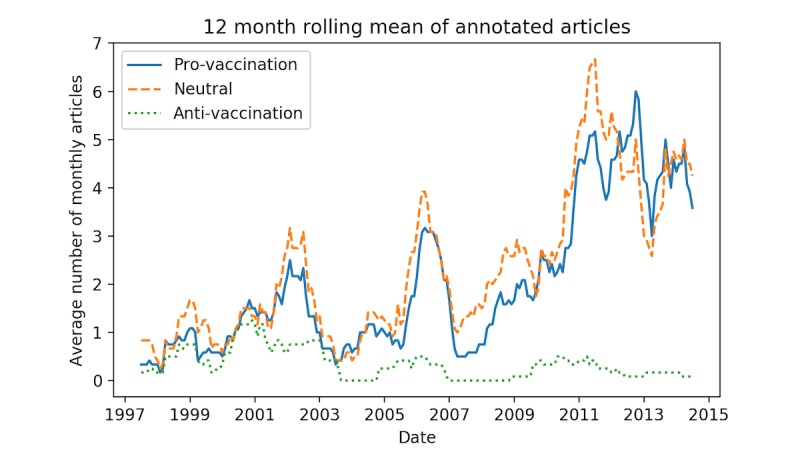
Vaccination attitude (stance) in media. For readability, we plotted a 12 months rolling mean. The rolling mean is calculated based on the number of articles published in a window of 6 months before and after a given data point.

Table 1 shows the number of news items in each category. The results clearly show low specificity of the retrieval method, with only 42.0% (681/1622) of the news items being relevant to the MMR vaccine. Figure 6 shows the distribution of each category during the study period. The peaks in provaccination and neutral information in 2002, 2006, and 2011 correspond to measles outbreaks. The majority of antivaccination news items occurred in the period 1997-2004. This coincided with the retracted study by Wakefield et al [5] published in 1998 that linked autism to the MMR vaccine. The antivaccination news items were primarily about the now falsified link between autism and MMR, but also about Danish court cases on allegations of adverse reactions to the MMR vaccine.

The first author annotated the complete dataset of 1622 news items. A random subset of 200 news items has been annotated by a second annotator, and the interannotator agreement was evaluated using the Cohen kappa coefficient to assess the quality of the annotation. For provaccination, the Cohen kappa coefficient is .54, for neutral it is .35, and for antivaccination, the score is .56. In other words, there is general agreement on provaccination and antivaccination, while less so for neutral.

### Relationship Between Media Coverage and Vaccination Activity

For the whole period 1997-2014, we found no significant correlations between vaccination activity and media coverage. This was the case both when we calculated the cross-correlation between MMR-1 vaccination activity and media coverage, and MMR-2 vaccination activity and media coverage, and similarly when using the annotated media data.

**Figure 7:**
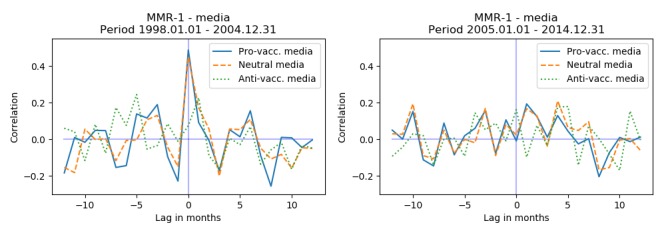
Cross-correlation for vaccination activity of MMR-1 and annotated media data for the 2 periods. (MMR: measles-mumps-rubella).

**Figure 8:**
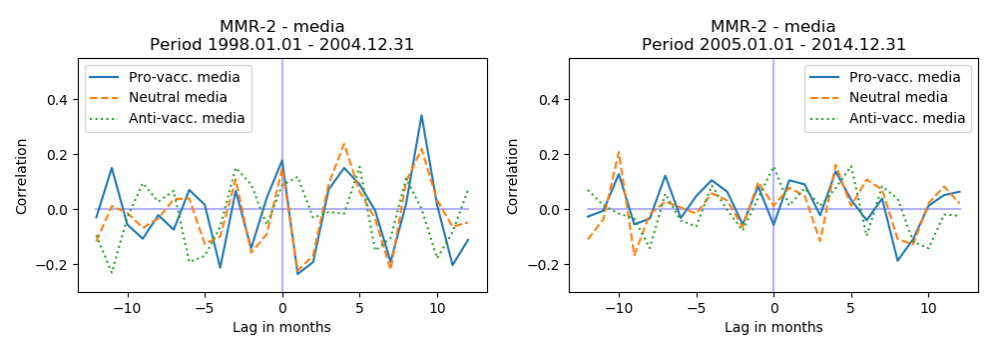
Cross-correlation for vaccination activity of MMR-2 and annotated media data for the 2 periods. (MMR: measles-mumps-rubella).

Most of the negative media coverage 65.3% (47/72) occurred in the period 1997-2004 (Figure 6). To assess if parents in this period were more susceptible to media influence than in the following period, we separated the dataset into 2: 1998-2004 and 2005-2014 (1997 is omitted because of the 12 months autoregressive models used to control for the seasonal changes and serial dependencies). Stratifying the data on these 2 periods, we found that for the period 1998-2004 there was a small but significant correlation at lag 0 between MMR-1 and all media (*r*=.32, *P*=.009). When using the annotated data, we saw that for the period 1998-2004 there was a statistically significant correlation between provaccination media and MMR-1 vaccination activity (*r*=.49, *P*=.004) and a statistically significant correlation between neutral media and MMR-1 vaccination activity (*r*=.45, *P*=.003). For MMR-2 we observed no significant correlation. Figures 7 and 8 show the cross-correlation at different lags for the 2 periods and 2 vaccines.

### Relationship With Measles Incidence

A possible confounder could be media coverage of measles outbreaks. To quantify this, we have analyzed the cross-correlations between vaccination activity and measles incidences, and between media coverage and measles incidence. We observe that the correlation between measles incidence and MMR-1 (*r*=.31, *P*=.005) was statistically significant at shift 1, meaning that an increase in measles incidence was followed the next month by an increase in MMR-1 vaccinations. For the media data, we found a statistically significant correlation at lag 0 between provaccination media and measles incidence (*r*=.38, *P*=.007). Though not statistically significant, the correlation between neutral media and measles incidence was also relatively high (*r*=.35). We observed no statistical correlations for MMR-2.

## Discussion

### Principal Results

Our study covered the period 1997-2014 and investigated the relationship between written media coverage and vaccination activity for the MMR vaccine in Denmark. Treating the whole period as 1 time series revealed no relationship between media and vaccination activity. However, the majority of antivaccination media coverage occurred in the beginning of the period (1998-2004). This represents a period where fear of adverse reactions to the vaccine was high, and the public discourse was tainted by the work of Wakefield [5] and others on the link between autism, as well as other diseases, and the MMR vaccination. During this period there is a statistically significant positive correlation between both provaccination media and vaccination activity for MMR-1 (*r*=.49, *P*=.004), and between neutral media coverage and vaccination activity for MMR-1 (*r*=.45, *P*=.003). In the period 2005-2014 we found no significant correlations. The observed correlations were small, indicating only a limited relationship between media coverage and vaccination activity. Additionally, we only observed the relationships for the first MMR vaccine, targeted the 15-month-old children. This could indicate that parents are more susceptible to media influenza when deciding on the first vaccine.

Analysis of the media coverage shows that peaks in provaccination and neutral media coverage often coincided with measles outbreaks. To quantify to what extent measles incidence is a confounder, we calculated the cross-correlation between media coverage and measles incidence. For provaccination media, there was a significant positive correlation of *r*=.38. This shows that there is a temporal relationship, but also that measles incidence does not fully explain the variations in the media coverage.

### Strengths and Limitations

The long study period of 18 years strengthens the research because the dynamics between media coverage and vaccination uptake could be studied both in a period with debate and in one without. The Danish vaccination register [16] ensures very reliable vaccination data on a per person level, which allows us to investigate timely changes in the vaccination activity. This is not possible with vaccination uptake data accumulated for each birth cohort.

There are some limitations to the study design. First, not all Danish media have been included, and information about news on radio and television are only present from May 2009 [17]. Additionally, social media have not been analyzed at all. However, we know from other studies on the relationship between social media and news media during a measles outbreak in the Netherlands, that the correlation between social media and news media is very high [27].

Another limitation is the annotation approach, specifically the threshold for when to rate a news item as relevant. Based on the subset of 200 news items annotated by a second annotator, it is evident that the threshold is unclear. This means that conclusions based on the absolute number of news items within a specific category can be questioned. For our analysis, this is not a problem, since we are using the Pearson correlation, which only considers changes relative to the mean. In other words, the information about the absolute number of news items is not used in the analysis.

Finally, it should be noted that we cannot make any statements on causality based on our results. Additionally, the general vaccination activity throughout the period is relatively stable, indicating a priori that external events only have a limited effect on the vaccination activity.

### Comparison With Prior Work

There has been previous work on analyzing the effect of media coverage on public behavior. In the 1970s during the US presidential elections, McCombs and Shaw [28] observed a correlation between people’s news consumption and their political opinions, which they defined as an agenda-setting effect. The agenda-setting effect depends on the issue at hand. If the issue affects people directly (eg, raising gas prices) the effect will be minimal; however, for more abstract issues (eg, trade deficits or balancing the national budget) the effect will be strong [29]. In our analysis of the media coverage, we saw that measles outbreaks are one of the strong drivers of provaccination and neutral media content, while antivaccination content is driven by fears of adverse reactions. We observe a significant correlation between media coverage and vaccination activity in the period with the most focus on adverse reactions. Analyzing the observation within the context of agenda-setting effects, one explanation could be that the risk of adverse reactions is hard to grasp, and the debate is often filled with discussions of abstract concepts such as relative risks of vaccination versus infection. We hypothesize that because the fear of adverse reactions is hard to relate to everyday life, people are more affected by the media when the discourse is dominated by safety concern, as we saw in the period 1998-2004. Another explanation for only observing a relationship in the period with a focus on safety maybe because it was a period where opposing views on vaccinations were expressed in the media. Similar observations have been made with respect to political debates [29], where a correlation between media coverage and people's opinions was observed for countries where the politicians did not agree, but no correlation was observed if the politicians agreed. In other words, when the media come to a consensus, their impact vanishes.

Related work more directly comparable to ours shows similar results. The effect of media coverage on vaccination uptake has been studied with respect to the influenza vaccine [12], HPV vaccine [10], and MMR vaccine [11,13]. Smith et al [11] focused on selective MMR nonrecipients, meaning children who received all recommended vaccinations except the MMR vaccine, and concentrated on media related to Wakefield et al [5] and its now discredited link between the MMR vaccine and autism. They concluded that there was a limited influence of mainstream media on MMR vaccinations in the United States. This fits with our results, where we also observe a limited effect. In a study by Mason and Donnely [13] they compare vaccination uptake in different areas of Wales for the period 1997-1998. They observed a lower vaccination uptake in areas where a series of anti-MMR vaccine articles had been published. Ma et al [30] concluded that media coverage together with recommendations from physicians was associated with increased influenza vaccination coverage in young children. Finally, Kelly et al [10] looked at the relationship between media exposure and knowledge about the HPV vaccine. They found that people exposed to health-related media had more knowledge about HPV than people with less exposure. These results indicate that, to some extent, there is an agenda-setting effect from the media on people’s vaccination behavior.

### Future Work

Vaccination programs are an essential part of most countries’ public health programs, and maintaining a sufficient vaccination coverage is high priority. With disinformation being used as a part of cyberwarfare [31], and the easy spreading of fake news [32,33] surveillance of traditional media and social media is an essential task for public health authorities. Digital media has made the publishing of information easy by both qualified and unqualified persons. The resulting variety of publication outlets of various authority make detailed surveillance an increasingly time-consuming task. One solution to this problem could be automation of the surveillance task. In our study, the crude retrieval method based on only a query showed very low specificity, only 42% (681/1622) of retrieved news items were judged relevant. Manual labeling was required to improve the specificity. This could be work we need to automate. Traditional sentiment detection will likely not suffice, since articles do not necessarily express negative views about the vaccine, but could, for example, emphasize benefits of “natural” immunization (ie, getting infected by measles). A related approach, namely stance detection [34], aims at automatically determining the stance expressed in ideological debates. Such approaches could potentially be used for detecting changes in attitudes expressed in the continuous stream of published media.

An important consideration when continuing the work on media monitoring is to assess to what extent the cost associated with the monitoring corresponds to the potential gain. Could changes in media coverage have been an early indicator of the reduced public trust in the MMR vaccine? And would this signal be strong enough to launch a proactive information campaign, potentially reducing vaccination distrust and the associated costs? The fact that media contains a potential for improved public health communication was illustrated in a study by Bahri et al [35], who showed that active monitoring of the HPV media debate and creation of derived questions could support proactive communication and preparedness. They estimated that the work corresponded to 49% of a full-time position. Extrapolating this to a full vaccination program corresponds to several full-time positions. This raises the question of whether new research within natural language processing, information retrieval, and machine learning could be used to automate this process and make it accessible at a low cost?

### Conclusion

This paper assesses the overall effect of media coverage on the rate of the MMR vaccination in Denmark during the period 1997-2014. The study shows that while for the whole period 1997-2014 there is no correlation between vaccination uptake and media coverage, there is a significant positive correlation in the period 1998-2004 between provaccination and neutral media coverage and vaccination activity for the first MMR vaccine. The period 1998-2004 was characterized by having both provaccination and antivaccination views expressed in the media. The results indicate 2 things: (1) the influence of media is stronger on parents when they are deciding on the first vaccine and (2) the effect of media coverage is stronger when it presents opposing viewpoints.

## Abbreviations

CRN: civil registry number
MMR: measles-mumps-rubella
